# Supplementary material for: Virus–prokaryote infection pairs associated with prokaryotic production in a freshwater lake
Source: mSystems. 2024 Jan 9;9(2):e00906-23. doi: 10.1128/msystems.00906-23 (PMC10878036; doi:10.1128/msystems.00906-23)
Supplement: Supplemental Figures — Figures S1-S5. [file msystems.00906-23-s0001.docx]

Supplementary material

**Virus–prokaryote infection pairs associated with prokaryotic production in a freshwater lake**

Shang Shen, Kento Tominaga, Kenji Tsuchiya, Tomonari Matsuda, Takashi Yoshida, and Yoshihisa Shimizu

Contact: Shang Shen

E-mail: s-shin@fc.ritsumei.ac.jp

This PDF file includes:

Fig. S1

Fig. S2

Fig. S3

Fig. S4

Fig. S5

**
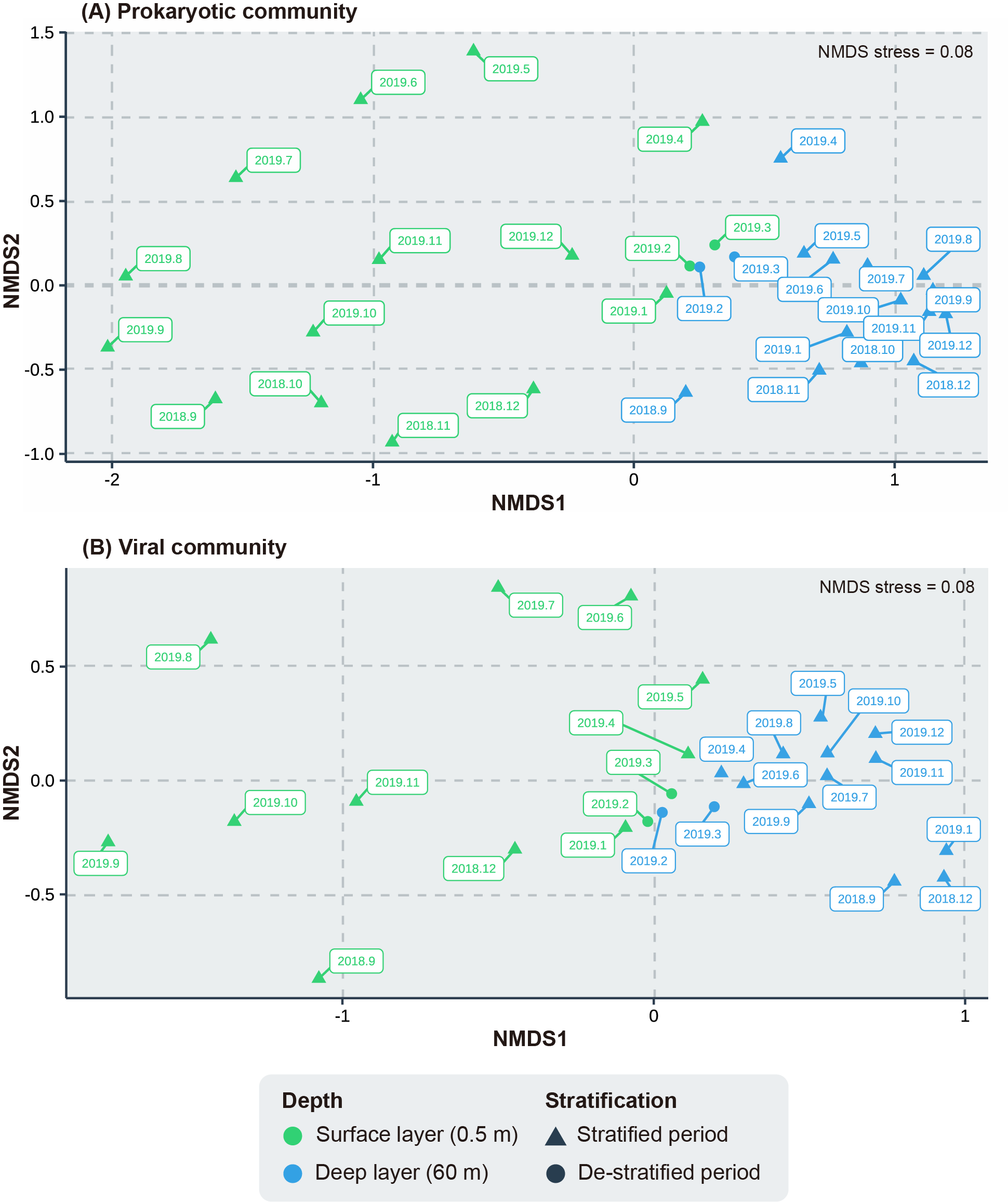
**

**Fig. S1.** Non-metric Multi-dimensional Scaling (NMDS) plots of (A) prokaryotic and (B) viral communities in the surface and deep layers during the study period

**
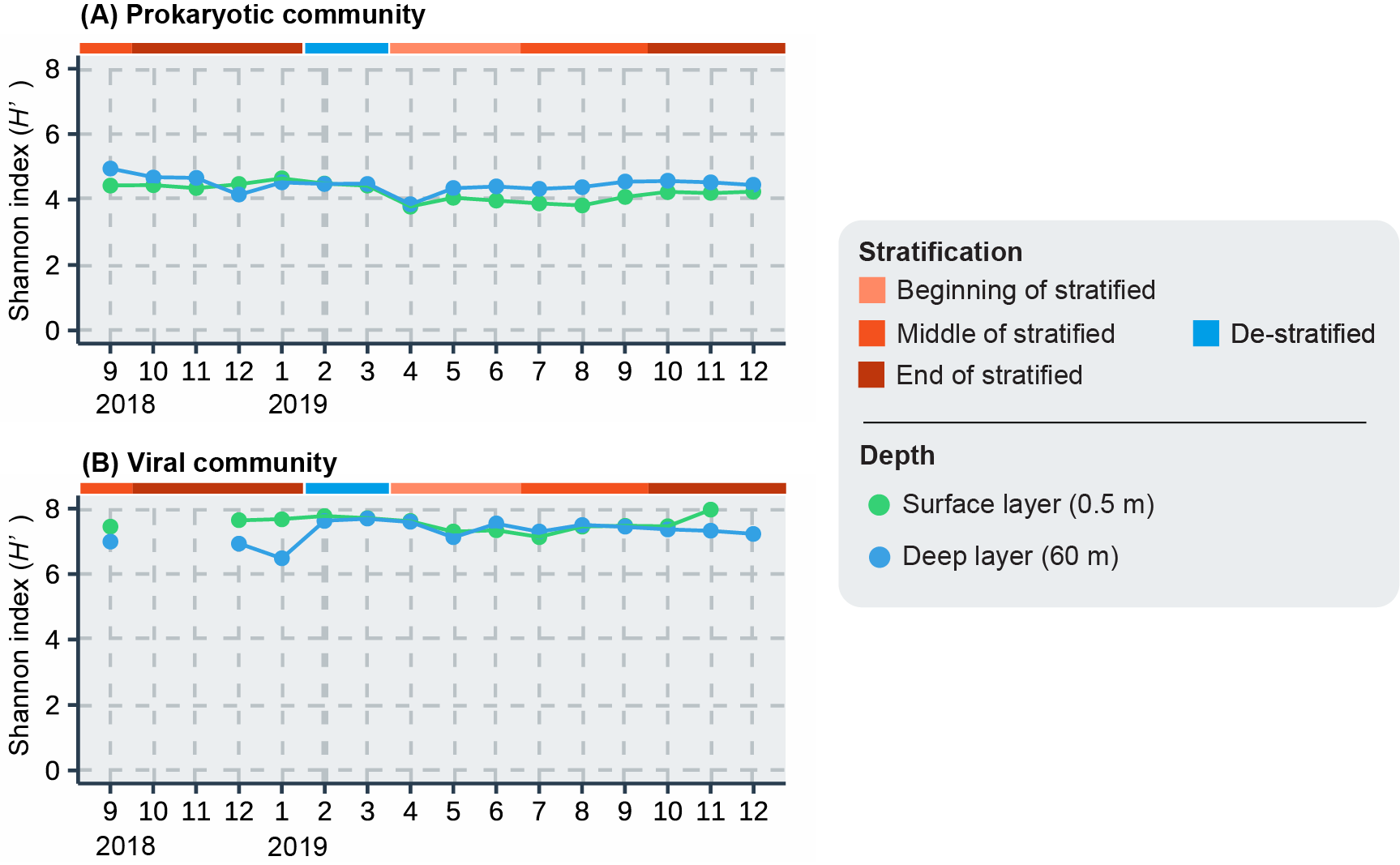
**

**Fig. S2.** Seasonal variation in the alpha diversity of (A) prokaryotic and (B) viral communities in the surface and deep layers

**
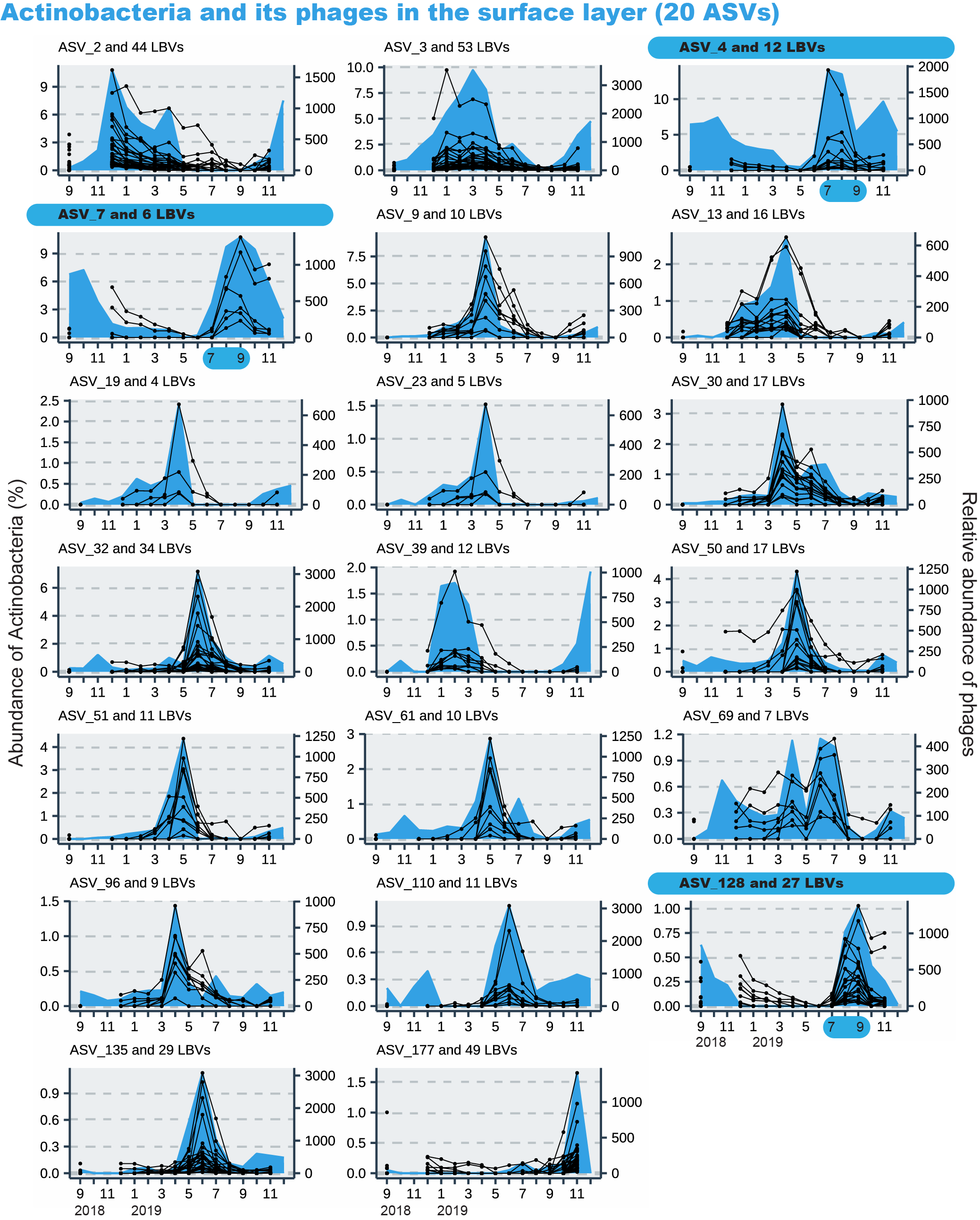
**

**Fig. S3.** Seasonal abundance of prokaryotic species (ASV) and viruses (LBV) co-occurring with the species in the surface layer. The subtitle of each panel indicates the ID of the ASV and the number of LBVs that co-occur with that ASV. Shaded subtitles indicate ASVs estimated to contribute to prokaryotic production (ASV_dominant_) in summer (July to September 2019, shaded months) and co-occurring LBVs. ASVs indicate amplicon sequence variants, and LBVs indicate Lake Biwa viruses.

**
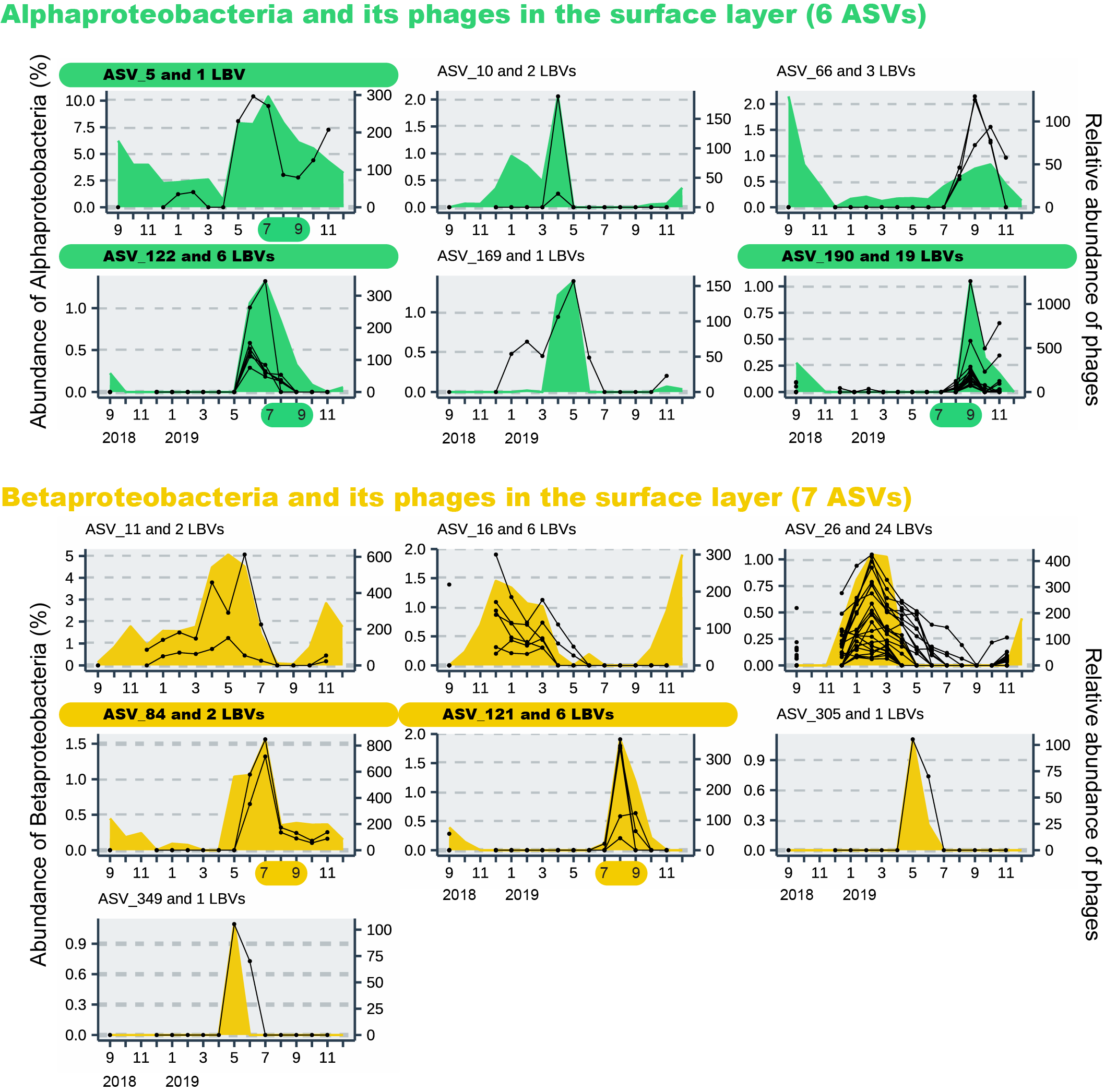
**

**Fig. S3 (continued)**

**
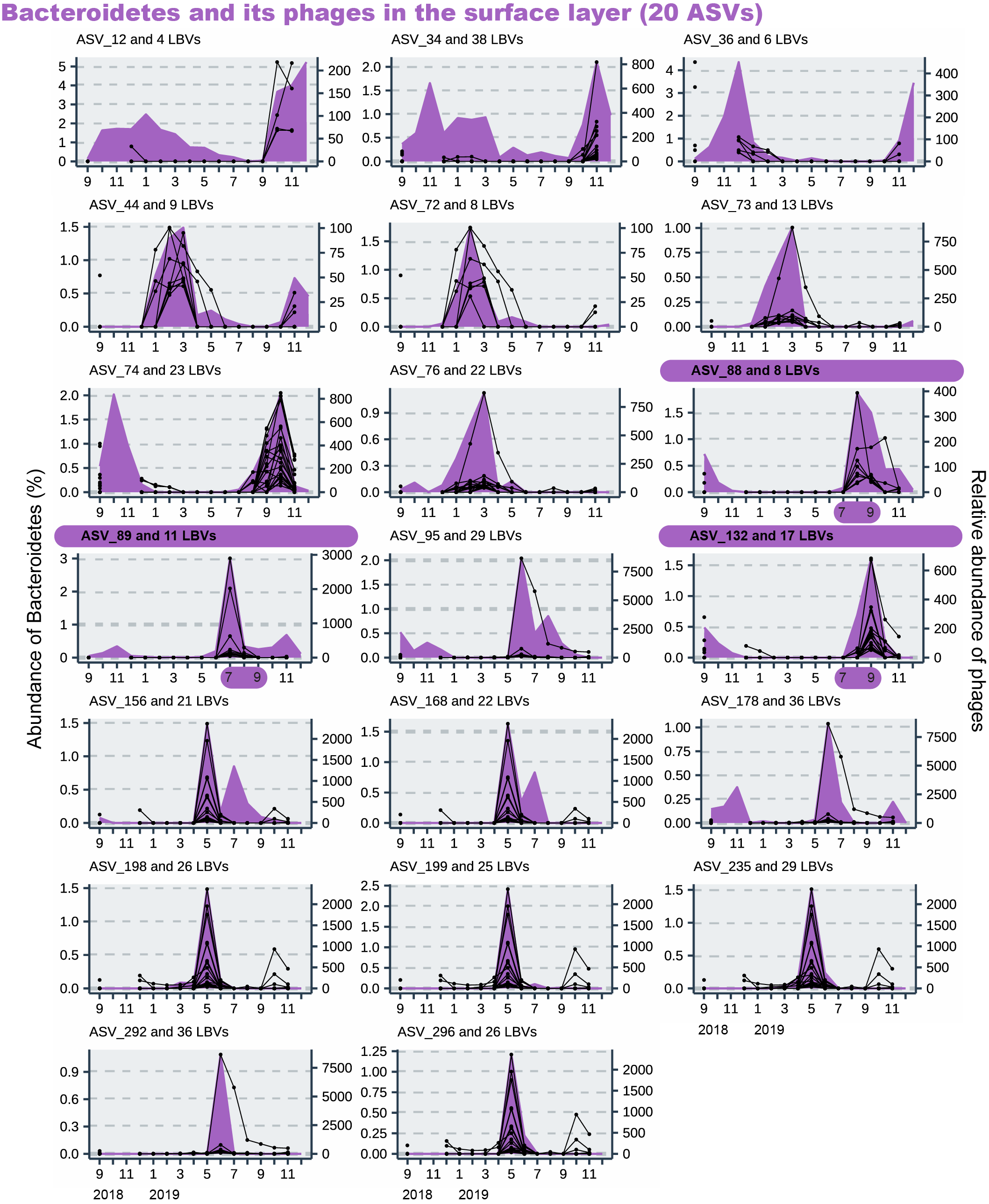
**

**Fig. S3 (continued)**

**
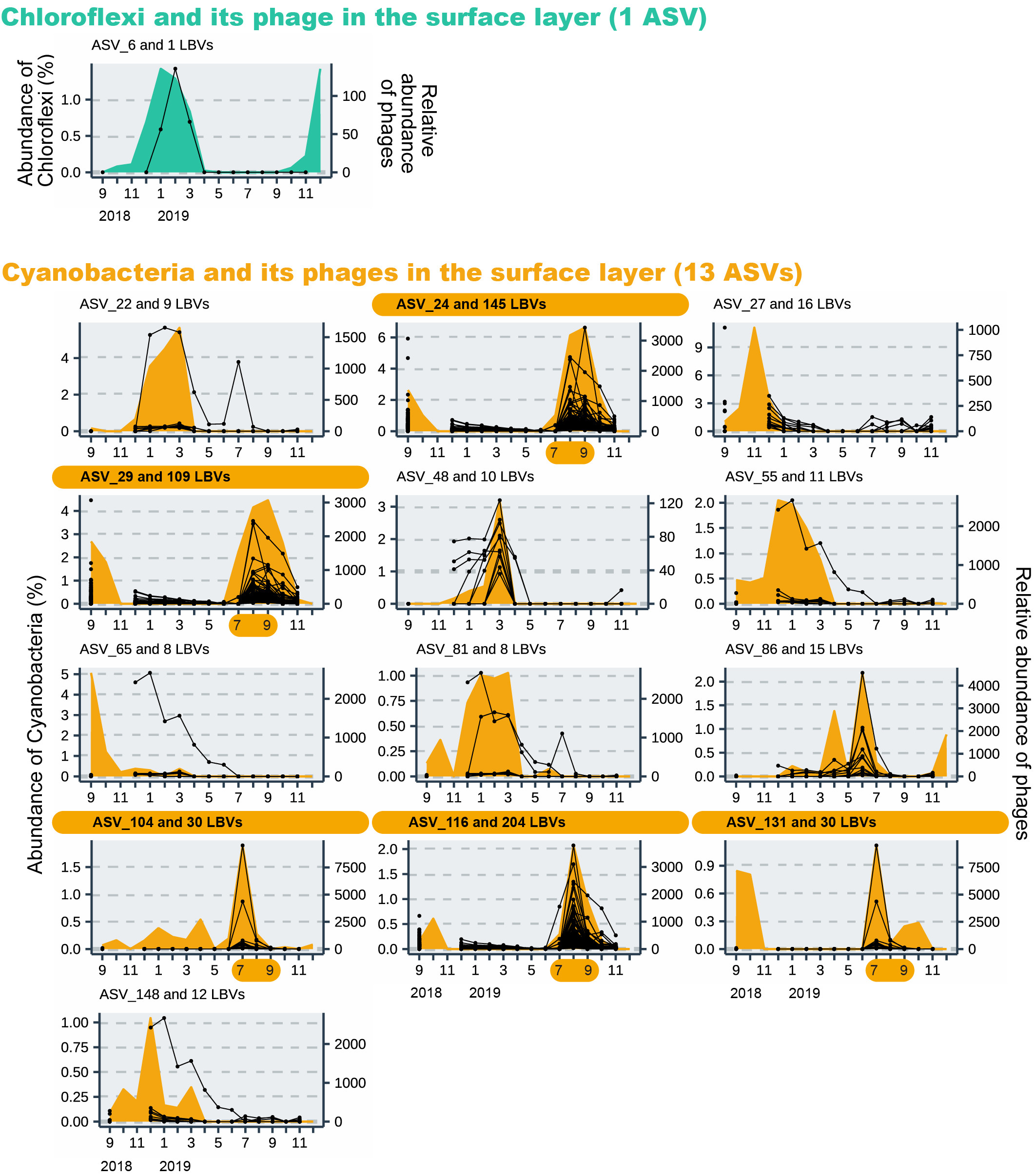
**

**Fig. S3 (continued)**

**
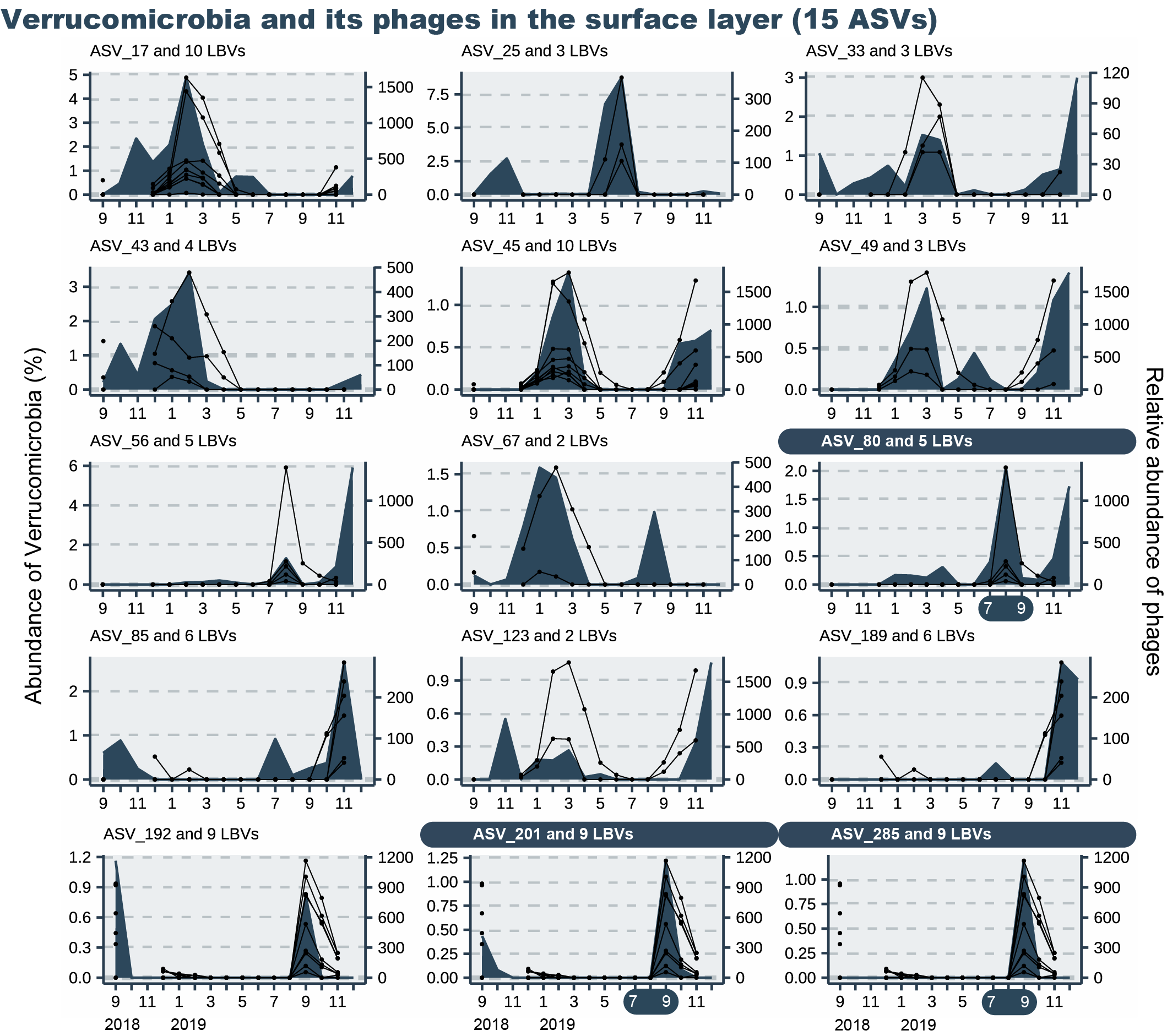
**

**Fig. S3 (continued)**

**
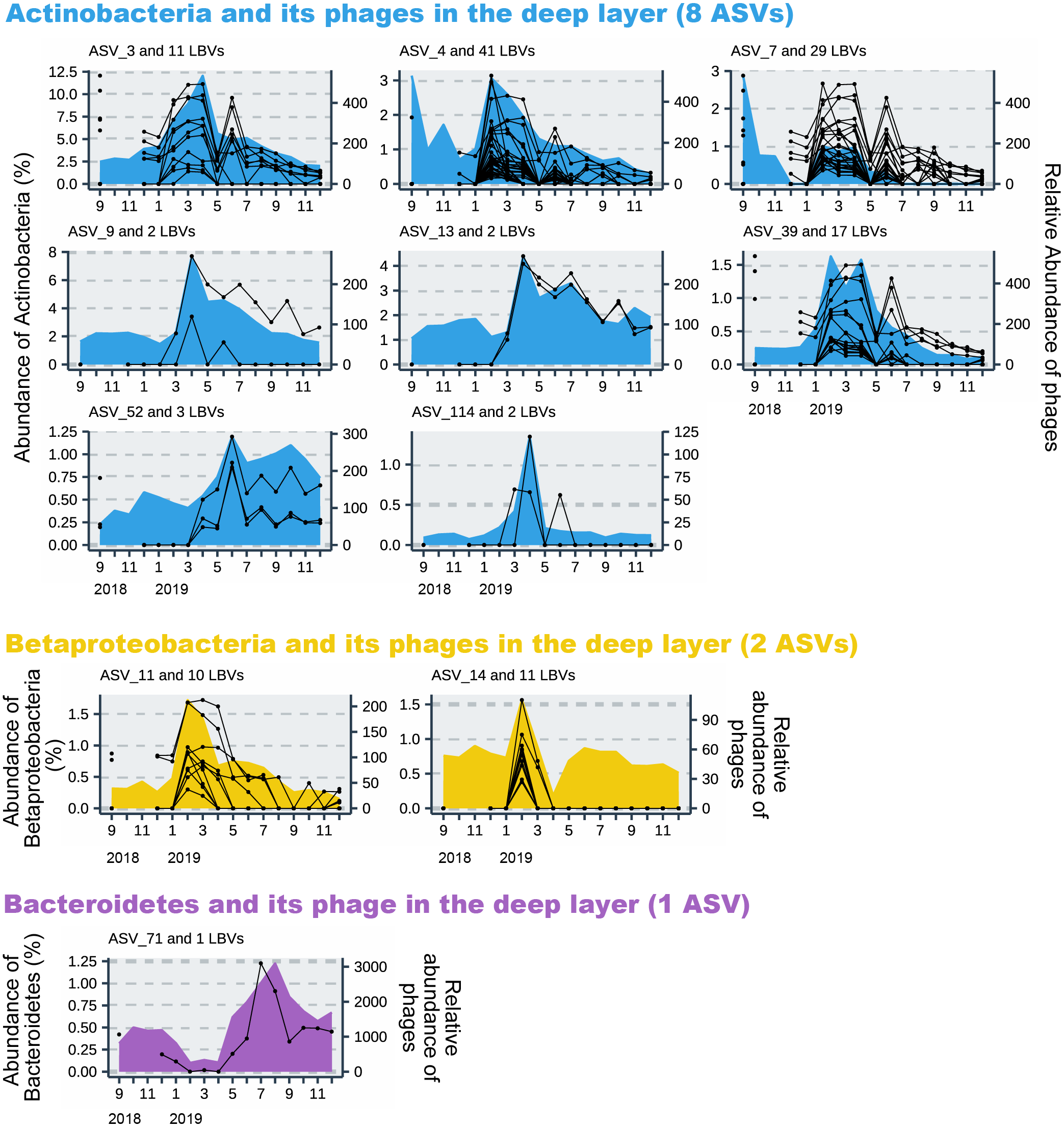
**

**Fig. S4.** Seasonal abundance of prokaryotic species (ASV) and viruses (LBV) co-occurring with the species in the deep layers. The subtitle of each panel indicates the ID of the ASV and the number of LBVs that co-occurred with the ASV. ASVs indicate amplicon sequence variants, and LBVs indicate Lake Biwa viruses.

**
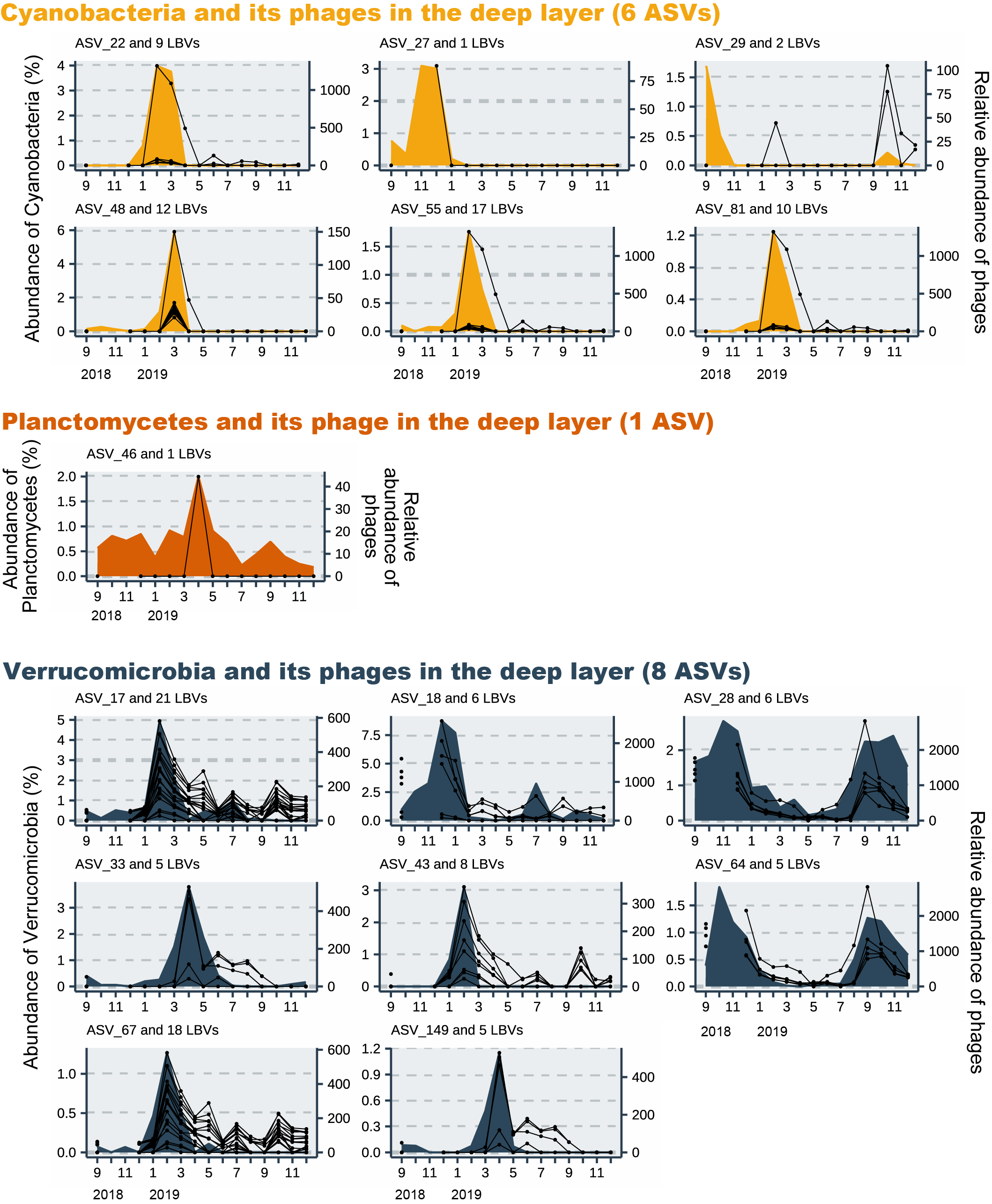
**

**Fig. S4 (continued)**

**
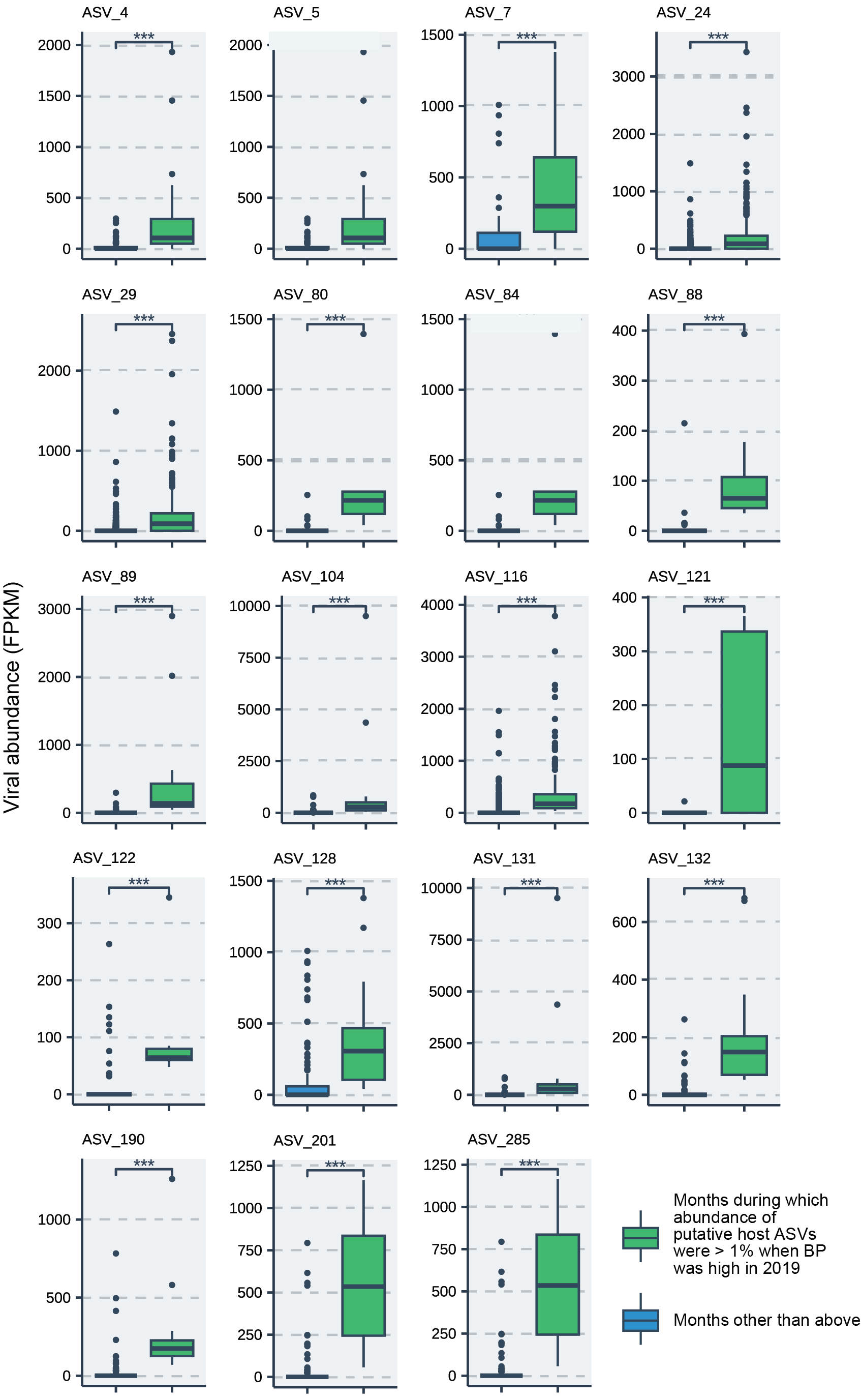
**

**Figure S5.** Abundance of LBVs co-occurring with ASV_dominant_ during which putative host ASV was > 1% when BP was high in 2019 and during other months. The Mann–Whitney U test was applied to test the statistical significance of an increase in LBVs abundance with an increase in the putative host ASV_dominant_. The results of the statistical analysis are shown in Table S7.
